# Medical Mistrust: A Concept Analysis

**DOI:** 10.3390/nursrep15030103

**Published:** 2025-03-17

**Authors:** Meghna Shukla, Marvin Schilt-Solberg, Wanda Gibson-Scipio

**Affiliations:** 1 Wayne State University College of Nursing, Detroit, MI 48202, USA; gibsonsc@wayne.edu; 2 School of Nursing, University of Michigan, Ann Arbor, MI 48109, USA; solberma@umich.edu

**Keywords:** medical mistrust, healthcare, concept analysis, nursing, provider

## Abstract

**Background:** The term “medical mistrust” has increased in literary usage within the last ten years, but the term has not yet been fully conceptualized. This article analyzes the usage of the term “medical mistrust” in the extant literature in order to articulate its antecedents, attributes, and consequences. The aim of this article is to provide a preliminary conceptual definition and conceptual figure for medical mistrust. **Methods:** Walker and Avant’s method of conceptual analysis was used to extract concept attributes, antecedents, and consequences and define empirical referents. The databases PubMed, CINAHL, Scopus, and PSYCinfo and the Google search engine were used. **Results:** Medical mistrust is a social determinant of health fueled by a fear of harm and exploitation and is experienced at both the interpersonal, intergenerational, and institutional levels, reinforced by structural racism and systemic inequalities. Medical mistrust is antedated by historical trauma, socioeconomic disparities, medical gaslighting, traumatic medical experiences, maladaptive health beliefs and behaviors, and individual minority identities and is transmitted intergenerationally and culturally. The consequences of medical mistrust include the underutilization of health services, delays in diagnosis and care, poor treatment adherence, poor health outcomes, negative psychological effects, and an increase in the uptake of medical misinformation and maladaptive health behaviors. **Conclusions:** The findings of this concept analysis have important implications for healthcare providers, healthcare systems, and researchers, as well as healthcare policy makers.

## 1. Introduction

### 1.1. A Definition of Medical Mistrust

Medical mistrust is a complex concept that generally refers to a lack of confidence in healthcare providers, institutions, and systems [1,2,3]. It encompasses skepticism towards advice or treatments, doubts about healthcare professionals’ motives, and fears of mistreatment or deception by the medical industry [4]. This concept has garnered attention due to its impact on patient outcomes and healthcare delivery. Individuals who have faced discrimination based on their race, ethnicity, or socioeconomic status are often more likely to harbor feelings of mistrust towards the system [5,6].

Medical mistrust significantly affects both individuals and healthcare institutions [2]. When patients lack confidence in their healthcare providers or hesitate to follow the recommended treatments, this can hinder effective disease management and result in poor health outcomes [4]. Moreover, individuals with negative perceptions of the healthcare industry may avoid seeking medical treatment even when facing urgent symptoms [7]. In some cases, this hesitancy stems from concerns about discriminatory treatment, further exacerbating healthcare disparities and undermining public health goals.

Medical mistrust also has economic consequences. Delaying or avoiding necessary care may result in higher medical costs for patients when their conditions worsen over time [8]. For healthcare institutions, mistrust can lead to decreased patient engagement and lower patient satisfaction rates, leading indirectly to financial losses resulting from a decreased patient volume.

In summary, understanding medical mistrust is crucial due to its far-reaching effects on individuals, healthcare systems, and society. Addressing the issue of medical mistrust can help identify contributing factors and develop interventions to reduce the healthcare disparities across communities.

### 1.2. The Difficulty Defining Medical Mistrust

Medical mistrust has been defined in various ways in the literature, largely due to its diverse manifestations among individuals and communities. Its definition is shaped by personal experiences, cultural backgrounds, and historical contexts [9]. For some, past negative encounters with healthcare providers fuel mistrust, while others develop skepticism through secondhand accounts of medical discrimination. Additionally, differing cultural perceptions of trust further complicate efforts to establish a universally accepted definition. Recognizing these diverse perspectives is essential to fully grasping the complexity of medical mistrust.

Shaughnessy and colleagues (2023) define medical mistrust as “a patient’s low level of confidence that their healthcare provider will act with benevolence during clinical interactions” [8]. While this definition emphasizes the importance of trust in patient–provider relationships, it does not capture the broader societal factors influencing medical mistrust [8]. Another common definition describes medical mistrust as “the suspicion held by individuals toward the motives underlying social situations involving health care delivery” [4,10]. This broader perspective acknowledges systemic influences beyond individual interactions. Common characteristics of medical mistrust include skepticism, fear, and uncertainty regarding the medical treatments suggested by healthcare providers [11]. By considering multiple definitions and perspectives, researchers can understand the complexities of medical mistrust better and work toward strategies to rebuild trust in healthcare systems.

### 1.3. The Purpose of the Analysis

#### Clarifying the Meaning of Medical Mistrust

The primary purpose of this concept analysis is to clarify the meaning of medical mistrust in healthcare settings, as it is a complex term with varying interpretations across individuals and communities. By examining its defining characteristics, contributing factors, and consequences, this analysis aims to establish a clearer conceptual map for understanding medical mistrust. A precise definition will also help differentiate it from concepts such as patient–provider communication and patient trust.

Medical mistrust has serious implications for both individuals and healthcare systems. Those who mistrust the healthcare system may delay or avoid seeking necessary treatment, leading to disease progression and increased healthcare costs [7]. Moreover, mistrust can contribute to poorer health outcomes and diminished perceptions of the quality of healthcare. It may also facilitate the spread of medical misinformation and encourage maladaptive health behaviors, such as the avoidance of healthcare and the utilization of non-prescribed medications to treat conditions.

Several key factors influence the development of medical mistrust, including historical events, socioeconomic status, cultural beliefs, and past personal experiences with the medical system. This concept analysis will explore how these factors interact in shaping attitudes and behaviors toward medical care. By providing a comprehensive understanding of medical mistrust, this study will lay the foundation for addressing its impact on health outcomes and healthcare systems.

## 2. Methods

Walker and Avant’s (2019) method of conceptual analysis was used to explore the concept of medical mistrust. Walker and Avant’s procedures for concept analysis include eight steps [12]:

1. Select a concept;
2. Determine the aims or purposes of the analysis;
3. Identify all uses of the concept that you can discover;
4. Determine the defining attributes;
5. Identify a model case;
6. Identify borderline, related, contrary, invented, and illegitimate cases;
7. Identify antecedents and consequences;
8. Define empirical referents.

As described, this analysis aimed to clarify the meaning of medical mistrust through the provision of a preliminary conceptual definition of the term.

### 2.1. The Search Strategy

This search was completed in two phases. First, a comprehensive literature review was conducted to gather relevant studies on medical mistrust from reputable sources, including academic journals, books, etc. The PICOS framework was applied to identify suitable articles for analysis. Searches were conducted in databases including PubMed, CINAHL, Scopus, and PsycINFO using keywords such as “medical mistrust”, “mistrust, “distrust”, and “healthcare disparities”, along with their combinations. Additionally, “medical mistrust” was searched as a compound phrase across databases. To minimize the publication bias and ensure a well-rounded analysis, gray literature—including expert editorials, news articles, blogs, dissertations/theses, and conference abstracts—was also reviewed [13,14]. In the case of this analysis, the exclusion of gray literature would have created an inaccurate description of the phenomenon. This step aimed to capture unpublished but relevant studies that may not be found in traditional academic sources.

The inclusion criteria for this concept analysis encompassed articles published in English from 1990 to 2024 to capture evolving societal attitudes and behaviors towards healthcare. Both qualitative and quantitative articles were incorporated to strengthen the analysis by providing diverse perspectives on medical mistrust. Blog posts were considered only if they focused on firsthand experiences of medical mistrust rather than general discussions of the topic. The analysis focused on studies conducted among diverse U.S. populations, particularly marginalized groups such as racial/ethnic minorities, individuals of low socioeconomic status, and those facing discrimination based on their gender or sexual orientation. This focus highlights the role of systemic inequalities in shaping experiences of medical mistrust in the United States. However, a potential methodological limitation is that the findings may not fully capture how medical mistrust manifests in other global contexts.

The exclusion criteria included non-English publications, studies conducted outside of the United States, opinion or editorial pieces, and articles focusing solely on provider–patient communication or trust in specific healthcare institutions. While these topics relate to medical mistrust, this concept analysis sought to examine its broader implications beyond individual interactions.

Two authors independently conducted the data extraction and analysis. Studies were analyzed for key themes related to medical mistrust, including historical events shaping mistrust in healthcare, socioeconomic disparities affecting access to care, cultural beliefs about health, and previous experiences with the healthcare system that influenced attitudes towards seeking care. The initial search yielded 866 articles, with 386 removed as duplicates. After screening their titles and abstracts, 192 articles were excluded. Following a full-text review, 95 articles met the inclusion criteria for the conceptual analysis. The articles were organized using Covidence digital software, and the research team collaboratively reviewed the data to ensure their consistency and accuracy.

**Figure 1:**
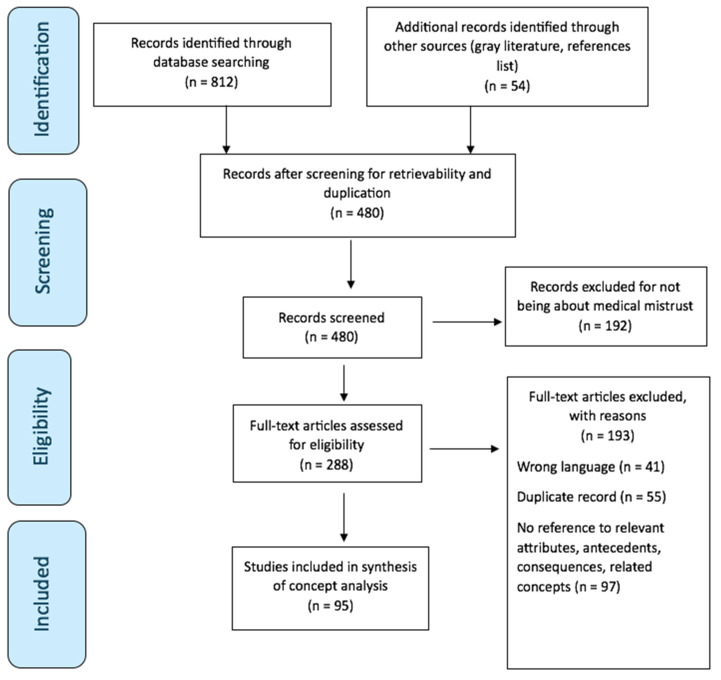
Flow chart of literature analysis.

To assess the studies’ quality, each article was evaluated based on its research objectives, study design, methodology, data analysis, results, and conclusions. This step ensured the inclusion of high-quality studies only. A content analysis and generalization techniques were applied to critically interpreting the data. Identified references were categorized into attributes, antecedents, and consequences for comparison. As Walker and Avant (2019) suggest, the eight steps of conceptual analysis can occur sequentially or simultaneously [12]; therefore, this study prioritized attributes, antecedents, and consequences before proceeding to the other steps to enhance the flow of the findings. Figure 1 depicts a PRISMA flow diagram of our search process.

### 2.2. Results of the Literature Review 

A significant body of biomedical literature has examined various dimensions of medical mistrust among different populations in the United States. The literature consistently indicates that medical mistrust is prevalent among racial and ethnic minorities, who experience healthcare disparities and systemic inequalities. These disparities shape individuals’ and communities’ interactions with medical institutions or professionals. Hence, medical mistrust has been described as “group-based”, reflecting a collective skepticism towards healthcare providers or institutions that have historically or currently mistreated specific groups. While this phenomenon can apply to any population, the extant literature has primarily focused on minority groups, including patients with HIV, African Americans (AAs), Latin/Hispanic Americans, and individuals who identify as LGBTQ [4,15,16,17,18,19,20,21,22,23,24]. Some studies suggest that medical mistrust functions as a protective measure and a form of “self-preservation” for minority groups [2,25].

Research consistently shows that people of color report higher levels of medical mistrust and conspiracy beliefs about the healthcare system compared to non-Hispanic whites [26,27,28,29]. Medical mistrust is prevalent within the AA community and is particularly pronounced due to historical mistreatment, dating back to slavery and unethical medical practices such as the Tuskegee Study of Untreated Syphilis in the Negro Male (TSUS) [30,31,32,33,34]. Given this history of medical exploitation and abuse, it is unsurprising that studies have consistently found elevated levels of medical mistrust among the AA community [15,16,17,18,19,20,21,22,23,24].

While trust and mistrust may seem like opposites, they are distinct concepts. Trust in the medical profession refers to a patient’s expectation that healthcare providers and institutions will act in their best interest [35]. In contrast, the extant literature has characterized medical mistrust by the suspicion that healthcare providers or institutions may engage in harmful practices [4]. Mistrust is not merely a lack of trust but rather a separate construct with unique emotional and behavioral implications. Trust plays a fundamental role in patient–provider relationships and influences healthcare behaviors such as seeking medical care [36], providing health information [37], consenting to treatment [37], adhering to medical recommendations [37], experiencing satisfaction with care [36], and maintaining continuity with the same provider [38]. The importance of trust in healthcare has been well documented, particularly in its role in patient–provider relationships and improving treatment adherence. Halbert and colleagues (2009) highlight that trust is influenced by factors such as race and prior exposure to healthcare providers [39]. Studies show that AAs report significantly lower levels of medical trust in healthcare providers and the healthcare system compared to those of European Americans [40,41,42,43,44,45].

Medical mistrust differs from medical trust in that mistrust is associated with negative emotions and should not be viewed as a point on a trust continuum ranging from low to high trust [4,46]. Trust, as it is traditionally defined and measured, does not account for suspicion of the healthcare providers or healthcare system. Higher levels of trust, however, have been linked to fewer emergency room visits, better treatment adherence (particularly in the management of hypertension and HIV) [47,48,49,50,51,52,53,54,55], and increased willingness to seek and stay in care [56,57,58,59].

## 3. Results

### 3.1. The Attributes of Medical Mistrust

Medical mistrust has been defined in various ways in the extant literature. This section identifies the key attributes of medical mistrust by examining the definitions and findings from relevant studies. In a concept analysis, defining attributes are essential characteristics that distinguish a concept from related terms and highlight its most commonly associated attributes [12]. Four key attributes of medical mistrust were identified in this concept analysis and are detailed below.

1. Interpersonal mistrust: suspicion of and skepticism towards healthcare providers’ intentions

A key attribute of medical mistrust is suspicion towards healthcare providers, characterized by doubts about their intentions, actions, or medical advice [4,34]. For example, a patient may question their doctor’s recommendation for a screening test due to concerns about potential harm or its necessity. This skepticism often arises from past negative experiences with healthcare providers that have left individuals feeling betrayed or disappointed [34,37]. Additionally, cultural beliefs related to disease etiology and treatment preferences can further influence mistrust, even among those who have not personally experienced mistreatment [46]. This attribute also encompasses patient doubts about the supportive nature of healthcare providers and the perception that providers do not advocate individuals like them [4]. This attribute was labeled as such to capture the doubts individuals have about medical professions’ motives and actions, shaped by personal experiences, cultural influences, and a perceived lack of provider support.

2. Institutional mistrust: a lack of confidence in healthcare institutions/systems

Beyond skepticism toward individual healthcare providers, institutional medical mistrust refers to a lack of confidence in larger healthcare entities, including hospitals, insurance companies, and pharmaceutical companies [60,61,62]. LaVeist et al. (2009) describes this mistrust as questioning the fairness, integrity, and ability of these institutions to provide unbiased care [63]. This skepticism is often rooted in historical injustices, such as the TSUS, which has contributed to long-standing distrust, particularly within minority communities [34]. Additionally, personal experiences with discrimination or mistreatment in healthcare settings further reinforce institutional mistrust, leading individuals to perceive these systems as untrustworthy or biased [64,65]. This attribute was labeled as such to distinguish broader skepticism toward medical organizations from interpersonal mistrust in individual providers. This term reflects the systemic dimension of mistrust, shaped by historical injustices, discriminatory practices, and concerns about institutional accountability.

3. Fear of exploitation and harm

Fear of exploitation and harm is a fundamental attribute of medical mistrust, reflecting concerns that individuals may be used for profit, research, or experimental treatments without their fully informed consent [34,64,66,67]. This fear often stems from historical incidents of unethical medical practices, particularly those targeting marginalized groups, such as women and other gender minorities, as well as vulnerable populations exploited in clinical trials [68]. Additionally, vicarious experiences, such as witnessing family members suffer from medical maltreatment, can reinforce this fear [69]. Concerns about unequal access to high-quality healthcare based on one’s socioeconomic status or race/ethnicity further contribute to the perception that the healthcare system prioritizes profit or institutional interests over patient well-being [25,70]. Since fear is central to medical mistrust, this attribute was labeled as such to highlight how anxiety over potential mistreatment or unethical practices fuels mistrust in healthcare systems. Medical mistrust, in this context, can be seen as a protective mechanism against anticipated harm, as the prevention of future harm, or as a coping strategy for dealing with discrimination and systemic inequities [64].

4. Mistrust about the medical care

Another defining attribute of medical mistrust is patient skepticism about the quality and safety of medical care [71]. This includes doubts regarding the validity and effectiveness of prescribed treatments, medical procedures, and healthcare techniques, with specific concerns about their potential side effects and adverse outcomes [72,73,74]. Individuals who experience medical mistrust often prioritize their psychological safety, seeking control over what enters their bodies, including medications, to avoid unnecessary or potentially harmful interventions [75]. This attribute was labeled as such to emphasize how uncertainty about the safety, efficacy, and necessity of medical treatments contributes to broader medical mistrust. The term reflects a patient’s desire for autonomy and caution when engaging with the healthcare system, often as a means of self-protection against perceived medical risks.

### 3.2. The Antecedents and Consequences of Medical Mistrust

#### 3.2.1. The Antecedents of Medical Mistrust

Walker and Avant emphasize the importance of identifying antecedents, which precede the concept, and the consequences which result from it [12]. The antecedents of medical mistrust are multifaceted, often stemming from historical instances of unethical research; discriminatory practices based on race, gender, or other minority statuses; and negative personal experiences within the healthcare system. Additionally, socioeconomic status, cultural beliefs, and past interactions with healthcare providers play a significant role in shaping medical mistrust.

1. Historical trauma

Historical trauma is a significant antecedent of medical mistrust, shaping individual’s perceptions and interactions with the healthcare system [2]. As discussed throughout this paper, unethical medical research and systemic mistreatment—such as the TSUS and the forced sterilization of minority women—have contributed a lasting impact on marginalized communities, fostering deep-seated suspicion and skepticism toward the medical system [7]. This distrust is not limited to past injustices but continues to be reinforced by institutional failures and disparities in healthcare treatment. For some populations, such as Indigenous peoples, medical mistrust is further compounded by intergenerational trauma stemming from colonization and systemic racism within settler societies. The historical exploitation of Indigenous communities in medical research and healthcare settings has reinforced negative perceptions of Western medicine and fueled ongoing reluctance to engage with the healthcare system [76].

Given that historical trauma has been a recurring theme throughout this paper, its role as a foundational antecedent of medical mistrust is well established. These past injustices serve as a critical backdrop for understanding why certain communities remain cautious about or resistant to medical interventions, even in contemporary healthcare settings

2. Socioeconomic disparities

Socioeconomic disparities are a key antecedent of medical mistrust, as limited access to high-quality healthcare services can erode trust in the medical system [77]. Individuals from low-income backgrounds often face significant barriers to obtaining adequate care, including financial constraints, a lack of health insurance, and the geographic inaccessibility of medical facilities. These systemic obstacles contribute to feelings of neglect and skepticism about whether healthcare institutions prioritize their well-being.

Research indicates that socioeconomic status—regardless of race/ethnicity—plays a critical role in shaping mistrust. Individuals with a lower income and lower levels of education consistently demonstrate higher levels of mistrust in healthcare providers and institutions compared to those with greater financial and educational resources [46,78,79]. Socioeconomic disparities serve as a critical antecedent of medical mistrust, as limited financial resources, lower educational attainment, and restricted access to high-quality healthcare contribute to skepticism about the medical system’s ability to provide equitable and effective care.

3. Personal experiences with discrimination

Personal experiences with discrimination—whether based on race, ethnicity, gender, or other identities—serve as a powerful antecedent of medical mistrust [80]. Encounters with bias and prejudice in everyday life shape individuals’ expectations of fairness and equity, influencing their willingness to trust people and institutions. Societal interactions and exposure to racism contribute to patterns of distrust toward authority figures and professionals, including healthcare providers [81]. These negative socialization experiences can, in turn, reinforce skepticism towards engaging with and reluctance to engage with the healthcare system [79,82].

Research has shown that medical mistrust significantly mediates the relationship between perceived discrimination and health behaviors such as medication adherence, treatment compliance, and preventive care utilization [21,83]. Individuals who have faced race-, ethnicity-, or gender-based discrimination may perceive the medical system as an extension of broader societal inequities, leading to heightened concerns about biased treatment, differential care, or potential harm in clinical settings.

4. Medical trauma

Medical trauma, whether experienced directly or vicariously, is a significant antecedent of medical mistrust. It can manifest as psychological or physical trauma, resulting from negative encounters with healthcare providers or medical institutions. Individuals may develop mistrust following traumatic medical experiences, including a poor bedside manner, a lack of transparency in decision-making, breaches of confidentiality, or perceived disrespect for their autonomy and physical well-being [84,85]. These distressing experiences can leave lasting psychological imprints, shaping how individuals engage with the healthcare system and whether they feel safe seeking medical care. Even those who have not personally experienced medical trauma may develop vicarious mistrust by witnessing negative medical encounters or hearing about them from family, friends, or community members.

5. Medical gaslighting

Medical gaslighting is a recognized form of medical injustice [86]. It occurs when a physician or another healthcare professional dismisses, trivializes, or invalidates a person’s symptoms, leading the patient to question their own experiences and well-being [87,88]. This can result in psychological distress, shame, depression, and anxiety about future medical encounters [88]. Consequentially, medical gaslighting is a significant contributor to the development and persistence of medical mistrust among individuals who experience medical gaslighting. Healthcare providers may exhibit medical gaslighting through dismissive attitudes, belittlement, condescension, or victim-blaming behaviors, particularly when they fail to take patient-reported symptoms seriously [86]. These interactions erode trust and discourage patients from seeking the necessary care, reinforcing their skepticism toward the medical system.

6. Health beliefs, health values, and health literacy/knowledge

Low health literacy has been identified as a contributor to medical mistrust [89]. Additionally, individuals who hold conspiracy-related health beliefs tend to exhibit higher levels of mistrust in the healthcare system [90,91]. Conspiracy beliefs may serve as a way to rationalize societal inequities, particularly among marginalized communities. Research indicates that people of color hold higher levels of both medical mistrust and conspiracy beliefs compared to non-Hispanic whites [24,25,26,27,28,29]. For example, persistent racial and ethnic disparities in HIV incidence and mortality have fueled HIV-related conspiracy theories within the Black/African American community. Common beliefs include claims that “HIV is a man-made virus” or that “AIDS was created by the U.S. government to control the Black population” [92]. These beliefs, shaped by historical and systemic inequities, reinforce medical mistrust and influence health behaviors.

7. Intergenerational transmission

Family systems theory suggest that families play a crucial role in shaping racial identity, socialization, and responses to racial interactions. This framework supports the existence of intergenerational medical trauma, which contributes to the transmission of medical mistrust across generations, influencing how individuals engage with healthcare systems. Additionally, families that minimize discussions about healthcare or devalue the importance of trusting providers and adhering to treatment plans further reinforce patterns of medical mistrust. These intergenerational influences shape perceptions of medical institutions and impact healthcare-seeking behaviors [69,93,94].

8. Cultural transmission

Medical mistrust can be a learned response to adversity among individuals with shared identities and cultural experiences [46,95,96]. It is often transmitted as a mechanism of empowerment and self-protection, particularly among those who perceive systemic barriers as working against their best interests [97,98]. This cultural transmission occurs within ethnic communities and peer networks and through social media [99].

9. Medical racism

The American healthcare system was founded on white supremacy, discriminatory medical practices, and systemic racism, with its roots tracing back to the antebellum slavery era [100,101]. In 2021, the Director of the Centers for Disease Control (CDC) declared medical racism a serious public health threat [102]. Discrimination based on race, ethnicity, gender, and other social identities significantly contributes to medical mistrust [63]. Implicit biases, or unconscious stereotypes about race, gender identity, weight, and sexual orientation, drive medical racism, leading to biased patient–provider communication and differential treatment [103,104]. These biases can result in healthcare professionals dismissing or invalidating patients’ symptoms based on their race, gender, ethnicity, or weight [22]. Perceived racism is one of the strongest correlates of medical mistrust [105]. Additionally, racial discordance between patients and providers—when patients receive care from providers of a different racial or ethnic background—further exacerbates medical mistrust [106,107,108].

10. Structural racism/structural inequities

Medical mistrust is closely linked to structural inequities that create unequal access to resources and opportunities across different population groups [2,101]. These inequities contribute to disparities in health outcomes and reinforce mistrust in the healthcare system [7,97]. For example, individuals from certain minority backgrounds often face structural financial and housing barriers through red-lining practices, thus limiting their geographical access to healthcare. This can foster the perception that the medical system does not prioritize their well-being, further deepening mistrust [109].

11. Identity status

Minority stress—stemming from one’s gender identity, sexual orientation, religious affiliation, or racial/ethnic minority status—is a significant contributor to medical mistrust [110,111]. Minority stress theory posits that stress disproportionately linked to minoritized identities leads to psychological distress, which in turn influences mistrust in medical institutions [112]. Studies show that sexual and gender minority youth report worse healthcare experiences and higher medical mistrust than their non-LGBT peers [113,114]. Additionally, gender identity, as categorized in studies using binary definitions, has been found to be significantly associated with medical mistrust. Across multiple studies, male gender was consistently linked to higher levels of medical mistrust than female gender [53,83,115,116,117,118]. Similarly, HIV-related mistrust and conspiracy beliefs were higher among males [115]. Racial and ethnic minority status remains a strong predictor of medical mistrust, as racial and ethnic minority groups frequently experience institutional discrimination in healthcare settings and beyond [2,65,119,120]. Race-related stress and perceived provider bias have been shown to contribute to medical mistrust, particularly among African Americans [116]. Discrimination in healthcare settings continues to perpetuate racial health disparities and reinforce mistrust among AA patients [44,121,122,123,124,125,126]. Religious differences between patients and physicians also influence medical mistrust, with some suggesting that addressing these differences may help reduce mistrust and improve patient–provider relationships [95,127].

#### 3.2.2. The Consequences of Medical Mistrust

##### Medical Mistrust Becomes a Social Determinant of Health, Leading to Health Inequities

1. The underutilization of health services

Medical mistrust can lead individuals to avoid seeking necessary medical care, resulting in delayed diagnoses, untreated conditions, and an overall diminished quality of life. Mistrust of healthcare providers acts as a significant barrier to utilizing healthcare services, discouraging routine and preventative care. Research has shown that medical mistrust is associated with higher rates of emergency department utilization, lower rates of preventative health services, and reduced engagement in health-seeking behaviors [1,3,105,128,129]. It thereby contributes to exacerbations in health disparities between racial and ethnic groups [24,65,105,122,125,130,131], specifically within the Black community [4,5,15,16,17,18,19,20,22,23,27,32,33].

2. A lack of adherence/compliance with treatment plans

Medical mistrust significantly impacts treatment adherence, as patients who mistrust their doctors or the recommended treatments are less likely to follow prescribed plans, resulting in ineffective disease management. Treatment adherence is a key clinical outcome frequently examined in research on medical mistrust. The World Health Organization (WHO) defines adherence as “*the extent to which a person’s behavior, including medication-taking, corresponds with agreed recommendations from a health provider*” [132]. Nonadherence is associated with increased morbidity and mortality, higher healthcare costs, and poorer clinical outcomes [132]. Studies have consistently shown that medical mistrust is linked to lower adherence in the management of chronic illnesses, such as hypertension [116] and HIV [22,83,115,118]. Participants with higher mistrust scores reported decreased adherence to treatment regimens, contributing to poorer health outcomes and exacerbating existing health disparities.

3. Delayed diagnosis and treatment

Medical mistrust can lead to delays in diagnostic testing and treatment initiation, resulting in poorer health outcomes. It is associated with delayed screenings for lung cancer, HIV, breast cancer, and colon cancer [15,22,64,133,134], as well as mental health evaluations, particularly among African Americans [135]. Mistrust also contributes to delays between diagnosis and treatment initiation [136,137].

4. Poor health outcomes

The fear of exploitation or unequal treatment—as shaped by past experiences and societal contexts—can lead individuals to avoid seeking medical care, directly resulting in poorer health outcomes [138]. These outcomes can be assessed both objectively through clinical measurements and subjectively through self-reports, though the congruence between the two may vary. Medical mistrust has been linked to worse outcomes in both types of assessments, including poorly controlled asthma [139], perceived diabetes control [140], and overall health-related quality of life [141,142].

5. Psychological effects

Medical mistrust has significant psychological impacts on patients. It is associated with lower satisfaction with healthcare providers and overall care experiences [5,21,26,27,108,143]. Medical mistrust also has a bidirectional relationship with emotional distress [144]. Chronic mistrust can lead to the persistence of distressing emotions, including feelings of inferiority, powerlessness, and a loss of faith in the healthcare system. Additionally, medical mistrust has been linked to anger in patients [145,146], as well as contributing to depression [147] and anxiety [148].

6. The uptake of medical misinformation

Race-based medical mistrust has been found to negatively influence patients’ beliefs about the necessity of medications and contribute to greater concerns about the use of medication [53]. The spread of medical misinformation through social media remains pervasive and continues to increase [149]. Many individuals turn to social media and public forums for health-related information [150,151,152]; however, these platforms often lack peer review and contain inaccurate or false information [153,154], which can lead to negative public health outcomes [155].

7. An increase in maladaptive and dysfunctional health behaviors.

Individuals who experience and endorse medical mistrust often engage in maladaptive health behaviors that negatively impact their well-being. These behaviors include avoiding healthcare encounters altogether or developing aversions to specific healthcare sites, procedures, or treatments. Medical mistrust also influences health communication behaviors, such as withholding information about risky behaviors or mental health issues, inaccurately completing medical history forms, and concealing symptoms during or before medical appointments [79,156,157,158]. These behaviors are often psychological protective mechanisms, intended to shield individuals from perceived medical harm. However, such behaviors worsen health self-management and contribute to increased health disparities [158].

#### 3.2.3. Synopsis

**Figure 2:**
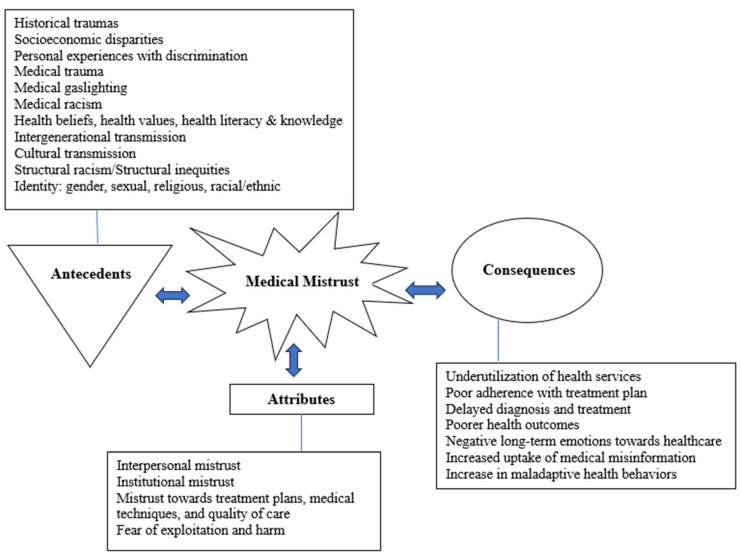
Conceptual model of medical mistrust.

In summary, the attributes, antecedents, and consequences of medical mistrust are critical factors that shape and influence an individual’s attitudes towards the healthcare system, as well as their health-related behaviors and outcomes. Further exploration of these components is essential for a more comprehensive understanding of this complex concept. Figure 2 is the proposed conceptual model of medical mistrust based on these findings.

Table 1 provides a structured framework for understanding medical mistrust, highlighting its four key attributes: interpersonal mistrust, institutional mistrust, mistrust in treatment and medical practices, and the fear of exploitation and harm. This table emphasizes that medical mistrust is not a singular phenomenon but a multidimensional construct shaped by various social, historical, and systemic factors. It also underscores the fluid nature of medical mistrust, wherein multiple antecedents (root causes) may contribute to its development, and any of the consequences (outcomes) may emerge as a result. Unlike traditional cause-and-effect models, this table allows for overlapping and intersecting pathways, acknowledging the complexity of how mistrust manifests in different contexts.

Table 2 further refines the concept by detailing the defining characteristics associated with each attribute of medical mistrust. This table synthesizes the key traits of mistrust identified in the literature, providing a more nuanced understanding of how mistrust presents itself at both the interpersonal and institutional levels. The attributes described in this table serve as the conceptual foundation for distinguishing medical mistrust from related but distinct constructs such as general skepticism towards, fear of, or distrust in other non-medical domains.

Table 3 outlines the various antecedents that contribute to the formation of medical mistrust, ranging from historical traumas and systemic inequities to personal experiences with discrimination and cultural transmission. By categorizing these antecedents, we provide a clearer understanding of the factors that contribute to the widespread nature of medical mistrust across different communities and populations.

### 3.3. Model Cases

This section presents hypothetical scenarios that exemplify the key attributes and consequences of medical mistrust, offering a clearer understanding of its impact on healthcare decision-making. The model and the related and contrary cases are informed by the findings from the literature and based on the results of our search. Through these model cases, we aim to illustrate the complexity and potential consequences of medical mistrust.

#### 3.3.1. Case One: Model Case

Maria, a 45-year-old Hispanic woman from a low-income background, was diagnosed with breast cancer. Throughout her life, she faced limited access to high-quality healthcare due to financial constraints. Her mistrust of doctors stemmed from past experiences where she felt ignored or mistreated by physicians. As a result, upon receiving her diagnosis, Maria became fearful of undergoing treatment, doubting its necessity or effectiveness. Despite the availability of medical interventions, Maria’s mistrust of the healthcare system led her to avoid follow-up appointments, opting instead to rely on home remedies recommended by friends. Unfortunately, this avoidance resulted in the progression of her cancer, ultimately leading to her death.

This case illustrates all of the attributes of medical mistrust. Maria’s interpersonal mistrust was shaped by negative past experiences with physicians, while her institutional mistrust stemmed from her limited access to quality healthcare services. Her mistrust in the treatment plan and quality of care, coupled with her fear of exploitation and harm, contributed to her decision to avoid medical interventions. Possible cultural influences, such as language barriers and customs, may have intensified her feelings of neglect. Additionally, social transmission—through stories from friends promoting alternative treatments—further reinforced her avoidance of formal healthcare.

#### 3.3.2. Case Two: Relevant Case

Robert is a 60-year-old African American man who has diligently managed his diabetes for over ten years. He adhered to his prescribed medication and followed a strict diet, showing trust in the medical advice he received. However, when his doctor informed him that he required open-heart surgery due to complications from uncontrolled diabetes, Robert’s confidence wavered. Robert’s hesitation and skepticism were influenced by his awareness of historical instances where African American individuals were subjected to unethical medical practices. Additionally, he had grown up hearing stories of racial discrimination within the healthcare system, which further reinforced his mistrust in medical professionals. Although an early intervention could have mitigated the severity of his condition, it was not until Robert had a near-death encounter that he hesitantly agreed to the surgery.

Robert’s case illustrates key attributes of medical mistrust. His knowledge of historical traumas and structural inequities and his fear of exploitation/harm significantly contributed to his delayed decision. However, his initial adherence to diabetes management suggests that his mistrust was context-specific, becoming more pronounced in circumstances involving invasive and life-threatening procedures. Therefore, while this case demonstrates key aspects of medical mistrust, it does not fully encapsulate the concept, classifying it as a relevant but not model case.

In both cases, we see interpersonal and institutional mistrust, as well as fear, significantly influencing the decisions patients make regarding their health outcomes. Moreover, past experiences with doctors and stories heard from others about mistreatment or discrimination serve as antecedents to medical mistrust. We also observe how these key factors interrelate with each other, resulting in an even deeper level of mistrust in the healthcare system, overall, leading to disastrous long-term effects [160]. From these cases, we also conclude that medical mistrust increases patient morbidity and mortality.

#### 3.3.3. Case Three: Contrary

Alex is an uninsured, low-income white man living in rural Appalachia who suffers from chronic back pain but avoids seeking treatment due to his mistrust in medical institutions. His mistrust stems from past experiences where his inability to pay for necessary procedures left him feeling marginalized and neglected. One day, while performing physical labor at his construction job, Alex’s pain became unbearable, forcing him to seek emergency room services. Tests revealed an acute herniated disc requiring surgery.

Despite his previous negative experiences, Alex’s visit to the emergency room turned out to be positive. The healthcare team treated him with respect and empathy, acknowledging his mistrust and addressing it through clear communication. They explained each step of the treatment process and took the time to answer his questions and concerns. This approach helped alleviate some of his apprehensions. The team’s commitment to providing high-quality care, regardless of Alex’s financial situation, allowed him to begin rebuilding trust in healthcare providers.

This case highlights how medical mistrust can be mitigated through proper communication and understanding from healthcare providers, even among individuals with pre-existing suspicion towards the medical system. It illustrates that while financial barriers and past experiences can contribute to medical mistrust, empathetic and respectful care can play a critical role in overcoming these barriers.

### 3.4. Empirical Referents

Empirical referents are measurable indicators that provide tangible evidence of a concept’s existence. Identifying these referents is the final step in a concept analysis [12]. In the context of medical mistrust, various measures have been developed to assess its presence and impact across different populations. These empirical referents are crucial for recognizing the extent of medical mistrust within society and informing the development of targeted interventions to address it.

LaVeist and colleagues (2000) developed the Medical Mistrust Index to measure general medical mistrust in hospital settings. This seven-item scale is based on a conceptual model that defines patient satisfaction as a function of individual predisposing characteristics—such as gender, educational attainment, and age—and experiential factors, including frequency of medical care encounters and attitudes toward the healthcare system [27]. The MMI assesses perceived racism, medical mistrust, and satisfaction with care within an institutional context [27]. It demonstrates acceptable internal reliability, with a Cronbach alpha of α = 0.76 [26,30,105]. Responses are measured using a Likert-type scale ranging from “strongly disagree” to “strongly agree”. The MMI has been used extensively in research involving diverse populations, including AA, Native American, and Latino communities [2].

Another widely used instrument for measuring medical mistrust among racial/ethnic minorities is the Group-Based Medical Mistrust Scale (GBMMS) [4]. The GBMMS is a 12-item scale utilizing Likert-type responses, ranging from 1 (strongly disagree) to 5 (strongly agree), with the total scores calculated by summing all item responses to yield a range of 12 to 60 [4]. Developed by Thompson and colleagues (2004), the GBBMS measured race-based medical mistrust as a barrier to breast cancer screening [4]. The scale includes statements reflecting mistrust in healthcare providers (e.g., “Doctors and health care workers sometimes hide information from my racial/ethnic group”), alongside reverse-coded statements (e.g., “Doctors have the best interests of people of my racial/ethnic group in mind”) [4]. The group-based component of the scale evaluates the tendency to mistrust individuals outside one’s ethnic group or systems perceived as non-representative due to a legacy of racism or unfair treatment [4]. The GBMMS is notable for being the first instrument to measure medical mistrust as an independent construct and for being the only scale that does not incorporate elements from Hall and colleagues’ Trust in Physicians Scale (TIPS), which conceptualizes trust through dimensions such as fidelity, competence, honesty, confidentiality, and global trust.

In comparing the two scales, it is notable that the GBMMS primarily focuses on personal experiences with a group referent, with only one item reflecting institutional systems. In contrast, the MMI contains items that address both institutional and personal systems, incorporating both general and personal referents. The MMI specifically asks participants about their beliefs regarding both medical personnel and broader medical organizations, with an emphasis on general perceptions (e.g., “people should” rather than “I should”). Additionally, the MMI examines provider competence, a dimension not addressed by the GBMMS.

In addition to developing these standardized measures, other research has explored how factors such as race/ethnicity, socioeconomic status, and personal experiences with discrimination influence medical mistrust within specific populations. For instance, Jaiswal (2019) explored how perceived racial/ethnic bias within healthcare settings impacted AA patients’ trust in their physicians [2]. Similarly, Bazargan and colleagues (2021) found that individuals from lower-income backgrounds reported higher levels of medical mistrust due to financial barriers to accessing care and perceptions of discrimination based on financial status [25]. However, studies on medical mistrust among sexual and gender minorities have primarily focused on HIV-related behaviors and their patterns of healthcare utilization. Research examining medical mistrust in other marginalized groups— such among immigrants, prisoners, and individuals with severe mental illness– remains limited. Additionally, the intergenerational transmission of medical mistrust is largely understudied.

It is also important to note that many of the existing studies have relied on selected subscales or single-item measures, which may limit the comprehensiveness of their findings regarding medical mistrust. Overall, these empirical referents highlight the multifaceted nature of medical mistrust and its impact on health outcomes among diverse populations. They also underscore the importance of culturally sensitive assessments, as different groups may experience mistrust in distinct ways. The use of standardized measures, alongside consideration of contextual factors, provides valuable insights into medical mistrust and can inform the development of targeted interventions. Incorporating these empirical referents into future research has the potential to reduce the mistrust of patients in healthcare systems, ultimately improving health outcomes. Table 4 summarizes these empirical referents.

## 4. Discussion

### 4.1. Definition

Based on this comprehensive conceptual analysis, we propose the following preliminary definition of medical mistrust:

“Medical mistrust is a social determinant of health fueled by an individual’s fear of harm and exploitation, presenting as suspicion of and lack of confidence in healthcare providers and systems, and is experienced at both the interpersonal, intergenerational, and institutional levels, reinforced by historical precedents and ongoing structural racism and systemic inequalities.” This definition highlights the multifaceted nature of medical mistrust and how it encompasses personal experiences, social determinants of health, and historical contexts.

### 4.2. Future Implications of Medical Mistrust

#### 4.2.1. For Future Research

Further research is needed to explore how medical mistrust impacts health outcomes and healthcare utilization among diverse populations. Studies that involve qualitative methods, such as in-depth interviews or focus groups with individuals who have medical mistrust, can provide valuable insights into the underlying factors influencing this phenomenon. Moreover, more culturally sensitive measures for assessing trust within specific populations should be developed to capture their experiences better. By understanding the complexity according to which social and ecological factors contribute to mistrust, nurse researchers can create targeted strategies to address mistrust and increase patient engagement in their care. Additionally, one key limitation of this review is that the included studies originated from the United States; further studies are needed to understand the transferability of the findings to other cultures and countries, where different unique factors may contribute to the phenomenon of medical mistrust. Understanding medical mistrust can help to inform future intervention studies that target this complex psychological phenomenon.

#### 4.2.2. For Healthcare Providers/Organizations

Healthcare providers must recognize the impact of past historical precedents and ongoing systemic racism and inequalities on patients’ perceptions in terms of trust in them. They must work towards building genuine relationships with marginalized communities in order to reduce medical mistrust. Patient-centered communication promotes attentiveness and empathy towards patients, especially those from disenfranchised minority groups, to improve patient–physician interactions and trust in healthcare professionals and the healthcare system [65]. Organizations should also strive to address socioeconomic disparities by providing equal access to high-quality care for all individuals regardless of their income levels or insurance status. By understanding the role of medical mistrust, nurses can provide better care for each patient and ensure the distribution of social justice by dismantling inequity not only in clinical practice but also in the fields of nursing research and education.

Additionally, healthcare providers must be aware of the potential implications for public health resulting from the behavioral consequences of medical mistrust. One potential behavioral consequence is that of nonadherence to medical treatment plans and medications. This can lead to the uptake of maladaptive behaviors, such as utilizing non prescribed, alternative substances to control chronic pain, for example. The implications that the adoption of these maladaptive behaviors hold for public health is the denigration of patient health outcomes, leading to increased disparate outcomes among marginalized and vulnerable populations.

#### 4.2.3. For Health Policy Makers

Addressing inequalities in healthcare access and developing policies that promote diversity and inclusivity within healthcare institutions could help build confidence in seeking healthcare services for those who may not have had positive experiences. Policies should be developed that aim to derail racist structural pillars and practices still in place today. The implementation of programs that are directed at decreasing the antecedents of medical mistrust discussed here will help empower patients to improve their relationship with their healthcare and ultimately improve their health outcomes.

## 5. Conclusions

In conclusion, this study sought to conceptualize medical mistrust and provide a definition using the methodology of Walker and Avant. Understanding medical mistrust is essential in promoting meaningful changes within our current healthcare framework, where health inequities continue to exist. It is essential for healthcare providers to recognize that patients who hold medical mistrust have legitimate antecedents that have led to its development. Addressing and disrupting the antecedents of medical mistrust will help bridge the gaps between patients and healthcare systems, leading to improvements in health outcomes and a decrease in the other consequences of medical mistrust.

## Figures and Tables

**Table 1:** Attributes, antecedents, and consequences of medical mistrust.

Attributes of Medical Mistrust	Factors Leading to Medical Mistrust (Antecedents)	Health and Behavioral Outcomes (Consequences)
Interpersonal mistrust (suspicion of and skepticism toward healthcare providers)	- Historical traumas - Medical racism - Personal experiences with discrimination - Medical gaslighting - Health literacy and beliefs - Intergenerational transmission	- Underutilization of health services - Negative psychological effects (anxiety, depression, anger, loss of faith in healthcare) - Poor communication between patients and providers
Institutional mistrust (distrust directed at healthcare systems)	- Structural racism and inequities - Socioeconomic disparities - Medical racism - Cultural transmission - Personal experiences with discrimination - Historical trauma	- Poor adherence to treatment plans - Delayed diagnosis and treatment - Avoidance of institutional healthcare settings
Mistrust in treatment and medical Practices (concern over medical techniques, quality of care, and provider decisions)	- Medical trauma - Personal experiences with discrimination - Medical gaslighting - Health literacy and conspiracy beliefs - Socioeconomic disparities	- Increased uptake of medical misinformation - Delayed or rejected medical interventions - Worsened disease progression due to late-stage diagnoses
Fear of exploitation and harm (concern about being deceived, harmed, or used unethically in medical settings)	- Historical traumas - Medical trauma - Medical gaslighting - Structural inequities - Identity-based discrimination (ethnicity, gender, sexual orientation, religion)	- Poor health outcomes - Avoidance of medical research and clinical trials - Increased reliance on alternative medicine and non-scientific treatments

**Table 2:** Manifestations of the attributes of medical mistrust.

Attributes of Medical Mistrust	Manifestations and Examples	Supporting References
Interpersonal mistrust (skepticism toward healthcare providers and their intentions)	Withholding information, decreased knowledge-sharing, inauthentic communication, and barriers to shared decision-making. Decreased empathy, respect, honesty, and integrity from providers. Uncertainty about providers’ motivations.	[4,34,36,37]
Institutional mistrust (in healthcare systems)	A perceived power imbalance, unresponsive or bureaucratic health systems, increased risks of medical errors, and decreased patient safety. Deceptive or unfair healthcare practices.	[27,34,61,62,63,64,65]
Mistrust in treatment and medical practices (concern over medical techniques, quality of care, and provider decisions)	Withholding treatment or providing it inconsistently, a perceived lower quality of care, concerns about a lack of transparency about medical procedures, and decreased medical supervision.	[25,34,64,66,67,68,69,70,71,72,73,74,75]
Fear of exploitation and harm (concern about being deceived, harmed, or used unethically in medical settings)	Concerns about unethical medical practices, including non-consensual procedures, being used as a “guinea pig” for experiments, and providers prioritizing personal gain over patient well-being. Concerns over physical harm, psychological distress, financial burden from unnecessary procedures, a loss of patient rights, and a loss of bodily autonomy.	[34,64,66,68,69]

**Table 3:** Manifestations of antecedents of medical mistrust.

Antecedents of Medical Mistrust	Manifestations and Examples	Supporting References
Historical Traumas	The legacy of unethical medical treatment (e.g., the mistreatment of African Americans, immigrants, and Indigenous populations)	[2,7,76]
Socioeconomic Disparities	Lower income, education levels, decreased healthcare access, housing insecurity	[46,77,78,79]
Personal Experiences with Discrimination	In both medical and non-medical settings	[21,79,80,81,82,83]
Medical Trauma	Physical, psychological, and financial harm from past medical experiences	[84,85]
Medical Gaslighting	Direct and vicarious experiences of patient concerns being dismissed	[86,87,88]
Medical Racism	Disparate treatment of racial and ethnic minorities in healthcare	[22,100,101,102,103,104,105,106,107,108]
Health Beliefs and Health Literacy	Misinformation, maladaptive health behaviors, conspiracy beliefs	[24,25,26,27,28,29,89,90,91,92]
Intergenerational Transmission	Passed-down experiences of mistrust, medical trauma	[69,93,94]
Cultural Transmission	Spread of mistrust via peer networks, institutions (workplaces, churches, extracurricular groups), and social media	[46,95,96,97,98,99]
Structural Racism and Inequities	Institutional, societal, and government-level discrimination in healthcare	[2,7,97,101,102,103,104,105,106,107,108,109]
Identity-Based Discrimination	Mistrust rooted in experiences related to LGBTQ identity, gender (e.g., female), racial/ethnic minority status (including immigrants), and religious affiliation	[2,30,44,53,65,83,95,110,111,112,113,114,115,116,117,118,119,120,121,122,123,124,125,159]

**Table 4:** Tools used to measure medical mistrust.

Tool	# of Items	Rating Scale	Aspects	Reliability (Cronbach’s Alpha)
Group-Based Medical Mistrust Scale (GBMMS)	12	5-point Likert Scale	Subscales are suspicion of healthcare providers, group disparities in healthcare, and lack of support from healthcare providers	0.83
Medical Mistrust Index (MMI)	7	4-point Likert scale	Mistrust of healthcare organizations and medical care systems	0.76

## Data Availability

Data sharing is not applicable. No new data were created or analyzed in this study.

## Public Involvement Statement

No public involvement in any aspect of this research.

## Guidelines and Standards Statement

This manuscript was drafted against the PRISMA guidelines utilizing the PRISMA-S extension checklist.

## Abbreviations

AA	African American
GBMMS	Group-Based Medical Mistrust Scale
MM	Medical mistrust
MMI	Medical Mistrust Index
